# Effects of vitamin K2 supplementation on atherogenic status of individuals with type 2 diabetes: a randomized controlled trial

**DOI:** 10.1186/s12906-021-03304-3

**Published:** 2021-05-01

**Authors:** Fatemeh Rahimi Sakak, Nazanin Moslehi, Hengameh Abdi, Parvin Mirmiran

**Affiliations:** 1 Nutrition and Endocrine Research Center, Research Institute for Endocrine Sciences, Shahid Beheshti University of Medical Sciences, No. 24, Shahid Arabi St, Yemen Blvd, Chamran Exp, Tehran, 1985717413 Iran; 2 Department of Clinical Nutrition and Dietetics, Faculty of Nutrition and Food Technology, National Nutrition and Food Technology Research Institute, Shahid Beheshti University of Medical Sciences, Tehran, Iran; 3 Endocrine Research Center, Research Institute for Endocrine Sciences, Shahid Beheshti University of Medical Sciences, Tehran, Iran

**Keywords:** Type 2 diabetes, Vitamin K, Menaquinone-7, Atherogenic status, Atherogenic index, Cardiovascular diseases

## Abstract

**Background:**

This study was aimed to examine the effects of vitamin K2 supplementation on atherogenic status, assessed by insulin resistance (IR)-related indexes, in patients with type 2 diabetes mellitus (T2DM).

**Methods:**

In this double-blind, controlled trial, 68 patients with T2DM on the oral glucose-lowering medications were randomly allocated into two groups receiving daily intakes of 360 μg MK-7 or placebo for 12 weeks. Eight different IR-related indexes were calculated at the baseline and end of the trial.

**Results:**

At the end of the study, atherogenic coefficient (mean ± SD: − 0.21 ± 0.45 vs. 0.02 ± 0.43; *p* = 0.043), triglyceride-glucose index (8.88 ± 0.55 vs. 9.23 ± 0.69; *p* = 0.029), and atherogenic index of plasma (0.37 ± 0.27 vs. 0.51 ± 0.24; *p* = 0.031) were significantly lower in the vitamin K2 group, compared to the placebo. However, after accounting for their baseline values, the differences were no more significant. No significant differences were observed in Castelli’s Ӏ and ӀӀ risk indexes, the ratio of triglycerides to high-density lipoprotein cholesterol, lipoprotein combine index, and the metabolic score for insulin resistance index between the two groups at the end of the study.

**Conclusions:**

Daily intakes of 360 μg vitamin K2 in the form of MK-7 for 12 weeks could not improve the IR-related indexes of Cardiovascular Diseases risk.

**Trial registration:**

The trial was registered on Iranian Registry of Clinical Trials registry (Trial ID. IRCT20190824044592N1) on 22 December 2019. The record can be found at https://en.irct.ir/trial/41728.

## Background

The burden of diabetes on public health and economic status will increase in the next few decades globally by raising the number of patients with diabetes [1]. These patients can live longer than before with medical science advancement, which increases the risk of diabetic-related complications [2]. Patients with diabetes are actually at risk of cardiovascular diseases (CVD), about four-time more than healthy ones, and the risk of cardiovascular deaths is also noticeably high [3]. Insulin resistance (IR) through glucose and lipid metabolism impairments is one of the leading causes of CVD risk [4]. The role of IR in endothelial dysfunction, plaque formation and progression, and vascular calcification is also indicated [4, 5]. Different indexes have been developed to assess IR indirectly, such as the atherogenic index of plasma (AIP), lipoprotein combine index (LCI), the metabolic score for insulin resistance index (METS-IR), and triglyceride-glucose index (TyG-Index). These non–insulin based indexes of IR can be easily determined by combinations of traditional risk factors of CVD, including fasting plasma glucose (FPG), lipid profiles, and body mass index (BMI). These indexes were associated with carotid atherosclerosis, vascular calcification, risk of CVD, and clinical outcomes, and therefore suggested to be useful for early detection of subclinical atherosclerosis [5–8]. The indexes are proposed to predict CVD events stronger than the routine individual lipid measurements [9].

A review article recently provided plausible evidence, suggesting the protective roles of vitamin K against vascular calcification through its anti-inflammatory effects and carboxylation of vitamin K-dependent protein (VKDP). Gamma carboxylation of Matrix Gla protein (MGP), a vitamin K-dependent protein, inhibits vascular mineralization. Of three forms of MGP identified in blood, dephosphorylated-uncarboxylated MGP (dp-ucMGP), a biomarker for vitamin K deficiency, was positively associated with vascular calcification, CVD events, and mortality [10]. However, a systematic review of the effects of vitamin K supplementation on clinical measures of vascular calcification, atherosclerosis, and arterial stiffness, reported that while the supplementation can decrease the levels of dp-ucMGP, the beneficial effects of vitamin K on reducing the risk of CVD is uncertain, according to the results of the current studies. Of the nine studies included in the systematic review, one conducted among patients with type 2 diabetes mellitus (T2DM) [11]. Vitamin K and CVD risks in patients with T2DM deserve further investigations because of the lower levels of vitamin K in the blood [12] and higher susceptibility to vascular calcification in this cohort of patients than healthy ones [13]. In a randomized clinical trial, we could find significant improvements in glycemic measures of FPG and glycated hemoglobin (HbA1c) in patients with T2DM with vitamin K2 supplementation in the form of menaquinone-7 (MK-7), compared to the placebo [14]. In this study, we aimed to investigate whether the MK-7 supplementation at a dose of 360 μg/d for 12 weeks could affect atherogenic status in patients with T2DM, assessed by eight different IR-related indexes, as secondary analyses.

## Materials and methods

### Participants

The original study design, which aimed to assess the effects of MK-7 on FPG and HbA1c in participants with diabetes, was reported in detail previously [14]. Briefly, in this randomized, double-blind, and placebo-controlled clinical trial, 68 patients with diabetes medicated with oral hypoglycemic drugs, aged 30–70, had at least one-year history of diabetes, and HbA1c between 6.5 and 10%, participated. Pregnant and lactating women, participants on insulin therapy or medications affecting the metabolism of vitamin K, and those with a history of chronic diseases such as heart, liver, kidney, and cancer were excluded.

### Ethical issues

This trial was designed according to the Declaration of Helsinki principles; the Ethics Committee of the Research Institute for Endocrine Sciences at the Shahid Beheshti University of Medical Sciences confirmed the protocols of the trial (IR.SBMU.ENDOCRINE.REC.1399.141). This study was also registered in the Iranian registry of clinical trials (Identifier number: IRCT20190824044592N1; date of registration: 22 December 2019). Before the study enrolment, the purpose and possible risks of the trial procedure were clearly explained to the participants, and then written informed consent was obtained from them.

### Trial design

After meeting the inclusion criteria, 68 eligible patients referred to three diabetic clinics in the north of Tehran were randomly assigned into the treatment groups of vitamin K2 (*n* = 34) or placebo (*n* = 34) using the method of block randomization with the block size of four and allocation ratio of 1:1. Each patient received a capsule container of vitamin K2 or placebo monthly in this trial. They were instructed to take two capsules of vitamin K2 (180 μg MK-7) or placebo per day after breakfast and dinner meals for 12 weeks. All participants were asked to avoid any dietary intake or physical activity changes throughout the intervention period and avoid consuming any other nutritional supplements. For designing a double-blinded trial, both vitamin K2 and placebo capsules were made in the same appearance and packaging by Arian Salamat Sina Pharmaceutical Company (Tehran, Iran). Microcrystalline Cellulose was used to fill the placebo capsules. At the Research Institute for Endocrine Sciences, a study leader generated the random assignment sequence, but she did not involve recruitment and trial procedures. The allocation was concealed by sequentially numbered of the capsules containers. A study executor enrolled the participants and assigned them to the intervention groups. Treatment assignments were blinded to all participants and the study executers until the statistical analyses were done.

At the end of each month during the study, participants received a new container of capsules and returned the previous one. Compliance with the treatment was checked by counting the remaining pills in each box. Moreover, we made weekly telephone calls to assess their adherence to the study protocol and the trial’s possible side effects.

### Biological variables analysis

After the 10-h overnight fasting, a 5 ml blood sample was collected at the beginning and end of the study at the laboratory of the Research Institute for Endocrine Sciences at Shahid Beheshti University of Medical Sciences. The enzymatic colorimetric method was used to measure FPG and Triglyceride levels by Parsazmun kits, Tehran, Iran. The enzymatic photometric method was used to assess the low-density lipoprotein cholesterol (LDL-C), high-density lipoprotein cholesterol (HDL-C), and total cholesterol levels by Parsazmun kits, Tehran, Iran. Intra-assay coefficients of variations (CVs) were 0.86% for FPG, 1.98% for total cholesterol, 1.46% for LDL-C, 0.61% for HDL-C, and 2.42% for triglycerides. The following indexes were calculated:

1. $$ Castell{i}^{\prime }s\  risk\ index\ I=\frac{Total\ cholesterol}{HDL-C} $$ [15]
2. $$ \frac{Triglyceride}{HDL-C} $$ [16]
3. $$ Atherogenic\ coefficient\ (AC)=\frac{Non\  HDL-C}{HDL-C} $$ [17]
4. $$ Castell{i}^{\prime }s\  risk\ \mathrm{index}\ \mathrm{II}=\frac{LDL-C}{HDL-C} $$ [15]
5. $$ Triglyceride- glucose\ index\ \left( TyG- Index\right)=\mathit{\ln}\frac{FPG\times Triglyceride}{2} $$ [18]
6. $$ Atherogenic\ index\ of\ plasma\ (AIP)=\mathit{\log}\frac{Triglyceride\ }{HDL-C} $$ [19]
7. $$ Metabolic\ score\ for\ insulin\ resistance\ index\ \left( METS- IR\right)=\frac{\left( LN\left(\left(2\times FPG\right)+ Triglyceride\right)\times BMI\right)}{\left( LN\left( HDL-C\right)\right)} $$ [20]
8. $$ Lipoprotein\ combine\ index\ (LCI)=\frac{Total\ cholesterol\times Triglyceride\times LDL-C}{HDL-C} $$ [21]

### Other clinical variables analysis

Data on disease duration, demographic variables, and drug medications were collected by a designed questionnaire. Weight and height were measured, and BMI was calculated [14]. Physical activity and dietary intakes were assessed at baseline and end of the trial using the short form of the International Physical Activity Questionnaires (IPAQ) and three 24-h food recalls, respectively.

### Statistical analysis

The study’s sample size was calculated based on the primary outcomes of the study [14]. The SPSS version 21 software was used to determine the statistical analyses. The normality of the variables was checked by the Kolmogorov-Smirnov test. Baseline characteristics of participants were compared between the two groups of the vitamin K2 and the placebo using the Student’s t-test for normally distributed variables, the Mann–Whitney U test for non-normally distributed variables, and χ2 test for qualitative variables. The mean value of each IR-related index of CVD risk after supplementation compared to the baseline values within each group using the paired sample t-test. The Student’s t-test was also used to compare each IR-related index’s mean value before and after supplementation between the two groups. Analysis of Covariance (ANCOVA) was used to adjust the baseline value of each outcome. The natural log-transformation was performed for non-normally distributed variables of AC and LCI before statistical analyses; mean and standard deviation (SD) values were reported for the natural log-transformed.

## Results

### Participants’ baseline characteristics

Participants were recruited from October 2019 to January 2020, and the follow-up was ended in April 2020. Of 68 participants included, 63 completed the study (32 patients in the vitamin K2 group, 31 patients in the placebo group); two individuals in the vitamin K2 group and three individuals in the placebo groups withdrew. The reasons for withdrawal were personal reasons (*n* = 3), immigration (*n* = 1), and bone surgery (*n* = 1). At the baseline, the mean age, diabetes duration, weight, BMI, and physical activity did not significantly differ between the two groups. Moreover, no significant difference was found in the number of females, smokers, and academic education between the vitamin K2 and the placebo groups. Baseline values of the indexes were not significantly different between the two groups (Table 1).

**Table 1:** Baseline characteristics of the participants^1^

Variables	Vitamin K_**2**_ group	Placebo group	***P***-***value***^2^
Number	32	31	–
Age, years	56.8 ± 7.34	58.2 ± 7.39	0.440
Disease duration, years	7.81 ± 4.27	9.68 ± 5.93	0.156
Female, n (%)	21 (65.6%)	18 (58.1%)	0.609
Smokers, n (%)	5 (15.6%)	2 (6.5%)	0.426
Academic education, n (%)	7 (21.9%)	14 (45.1%)	0.201
Weight, kg	7.29 ± 14.7	74.9 ± 16.7	0.607
Body mass index, kg/m^2^	27.9 ± 4.90	27.5 ± 3.60	0.729
Physical activity, Met-min/week	425 (209–679)	834 (240–1386)	0.063
Atherogenicity index			
*Castelli index Ӏ*	3.33 ± 0.79	3.65 ± 0.89	0.133
*Triglycerides / HDL-C ratio*	1.33 ± 0.85	1.64 ± 1.06	0.194
*AC*^3^	−0.17 ± 0.48	0.01 ± 0.44	0.124
*Castelli index ӀӀ*	1.68 ± 0.48	1.80 ± 0.51	0.308
*TyG-Index*	9.02 ± 0.56	9.23 ± 0.64	0.172
*AIP*	0.40 ± 0.27	0.51 ± 0.24	0.109
*METS-IR*	43.9 ± 9.09	45.1 ± 7.14	0.576
*LCI*^3^	1.95 ± 0.37	2.03 ± 0.40	0.382

*AC* Atherogenic Coefficient, *AIP* Atherogenic Index of Plasma, *LCI* Lipoprotein Combine Index, *METS-IR* Metabolic Score for Insulin Resistance, *TyG-Index* Triglyceride glucose Index

^1^Data are presented as mean ± SD, median (quartile 1-quartile 3), and number (%)

^2^Based on T-test for normally-distributed, Mann-Whitney U-test for non-normally distributed and chi-square for categorical variables

^3^Natural log-transformed values are reported

Of the participants, 64.7% were treated with lipid-lowering drugs, 97% were treated with metformin, and 19.2% were treated with the sodium-glucose co-transporter-2 (SGLT2) inhibitors. At the baseline, the frequency of lipid-lowering and oral hypoglycemic drugs did not significantly differ between the two groups.

Physical activity at the end of the trial and dietary intakes at baseline and end of the study were not significantly different between the two groups (data were not shown).

### Effects of vitamin K_2_ on atherogenic status

Compared to the baseline, a marginally significant reduction was observed in the METS-IR in the vitamin K2 group (mean change from baseline: − 0.80 ± 2.24; *p* = 0.052). None of the other indexes changed significantly compared to the baseline within groups. At the end of the study, AC (− 0.21 ± 0.45 vs. 0.02 ± 0.43; *p* = 0.043), TyG (8.88 ± 0.55 vs. 9.23 ± 0.69; *p* = 0.029), and AIP (0.37 ± 0.27 vs. 0.51 ± 0.24; *p* = 0.031) were significantly lower in the vitamin K2 group compared to the placebo group, but the differences became non-significant after adjusting for their baseline values. The other indexes were not significantly different between the two groups at the end of the study (Table 2).

**Table 2:** The effects of vitamin K2 supplementation on atherogenic indexes^1^

Variables	Vitamin K2 group (***n*** = 32)	Placebo group (***n*** = 31)	Between group ***p-value***^**3**^	Adjusted ***p-value***^**4**^
**Castelli index Ӏ**^**2**^				
After 12-week	3.29 ± 0.75	3.67 ± 0.91	0.073	0.333
Mean change from baseline	– 0.04 ± 0.45	0.02 ± 0.55	0.657	–
Within group *P*-value^5^	0.657	0.834	–	–
**Triglycerides / HDL-C ratio**				
After 12-week	2.81 ± 1.94	3.74 ± 2.25	0.083	0.252
Mean change from baseline	−0.10 ± 0.71	− 0.01 ± 0.51	0.570	–
Within group *P*-value^5^	0.442	0.925	–	–
**AC**^**6, 7**^				
After 12-week	−0.21 ± 0.45	0.02 ± 0.43	0.043	0.167
Mean change from baseline	−0.03 ± 0.25	0.01 ± 0.21	0.407	–
Within group *P*-value^5^	0.440	0.709	–	–
**Castelli index ӀӀ**^**8**^				
After 12-week	1.69 ± 0.41	1.88 ± 0.54	0.132	0.264
Mean change from baseline	0.02 ± 0.30	0.07 ± 0.38	0.506	–
Within group *P*-value^5^	0.777	0.297	–	–
**TyG-Index**				
After 12-week	8.88 ± 0.55	9.23 ± 0.69	0.029	0.084
Mean change from baseline	−0.14 ± 0.46	0.0005 ± 0.42	0.205	–
Within group *P*-value^5^	0.091	0.995	–	–
**AIP**				
After 12-week	0.37 ± 0.27	0.51 ± 0.24	0.031	0.135
Mean change from baseline	−0.03 ± 0.15	0.003 ± 0.12	0.325	–
Within group *P*-value^5^	0.245	0.901	–	–
**METS-IR**				
After 12-week	43.1 ± 8.42	45.2 ± 7.29	0.292	0.067
Mean change from baseline	−0.80 ± 2.24	0.15 ± 2.30	0.101	–
Within group *P*-value^5^	0.052	0.719	–	–
**LCI**^**7**^				
After 12-week	1.91 ± 0.34	2.04 ± 0.43	0.194	0.315
Mean change from baseline	1.34 ± 0.57	1.54 ± 0.53	0.167	–
Within group *P*-value^5^	0.391	0.842	–	–

*AC* Atherogenic Coefficient, *AIP* Atherogenic Index of Plasma, *LCI* Lipoprotein Combine Index, *METS-IR* Metabolic Score for Insulin Resistance, *TyG-Index* Triglyceride glucose Index, *HDL-C* High-density lipoprotein cholesterol

^1^Data are presented as mean ± SD

^2^Total cholesterol / HDL-C ratio

^3^Based on independent sample T-test

^4^Based on Analysis of Covariance (ANCOVA)

^5^Based on paired sample T-test

^6^Non-HDL-C / HDL-C ratio

^7^Natural log-transformed values are reported

^8^LDL-C/HDL-C ratio

### Sensitivity analysis

Glucose-lowering medications of eight participants were changed during the study, all of whom were in the placebo group (drug dose reduction (*n* = 4), dose increment (*n* = 3), added insulin (*n* = 1)). Lipid-lowering medications did not change during the study. However, no remarkable differences were observed in our findings after re-analyzing the data by excluding the eight participants.

### Compliance and tolerability

The study’s compliance was 95.9 ± 5.99%; none of the participants consumed less than 70% of the study capsules. Vitamin K2 and placebo capsules were both well-tolerated, and none of the participants reported any side effects related to the capsules’ consumption.

## Discussion

After a 12-week supplementation of 360 μg/day MK-7, AC, AIP, and TyG index were significantly lower in the vitamin K2 group than the placebo group, but the differences did not remain significant after accounting for their baseline values. No significant differences were observed in the other indexes within and between groups.

Vitamin K involves different physiologic activities by promoting the carboxylation process of VKDP as a co-factor for the enzyme of gamma-carboxylase. Gamma-carboxylation of the proteins makes them biologically active [22]. The most well-known VKDP produced by smooth muscle cells is MGP that inhibits vascular calcification. A higher circulatory level of dp-ucMPG, the best vitamin K insufficiency marker, has been related to a higher risk of vascular calcification, cardiovascular events, and CVD mortality [10, 23]. The anti-inflammatory effects of vitamin K also increase the possibility of its contribution to cardiovascular health [10, 22]. Higher intakes of dietary vitamin K have been associated with the lower risk of total coronary heart disease based on the pooled data from prospective studies on associations between dietary intakes of vitamin K1 (*n* = 4) and K2 (*n* = 2) and cardiovascular events. However, no significant association between dietary intakes of vitamin K and CVD mortality and stroke was found [23]. A small number of clinical trials examined the effects of vitamin K supplementation on CVD markers [11]. Recently, a study systematically reviewed clinical trials’ findings for the effects of vitamin K supplementation on the clinical proxy of CVD. Vitamin K supplementation could reduce dp-ucMPG significantly compared to the placebo in 7 out of 9 studies, but the effects of the supplementation on vascular calcification, atherosclerosis, and arterial stiffness were not convincing due to the scarcity of the studies [11].

Circulatory vitamin K1 was lower in patients with T2DM compared to non-diabetic individuals. The lower vitamin K status was also correlated with higher vascular inflammation as measured by *monocyte chemoattractant protein-1* (MCP-1) [12]. Increased vascular calcification is also documented in patients with T2DM due to their inflammatory state. Moreover, inadequate control of glycemia, increased IR, and kidney disease occurrence accelerates the calcification process [24]. Increased risk of CVD, peripheral arterial disease, and heart failure in a prospective study with a median duration of 11.2 years were reported for patients with T2DM with low vitamin K status, as assessed by plasma dp-ucMGP [25]. To the best of our knowledge, the effects of vitamin K supplementation on the vasculature in patients with T2DM has been investigated only in one clinical trial. The study examined the effect of MK-7 supplementation on the clinical surrogate of artery calcification measured by computed tomography [26]. Based on the findings, six-month supplementation of 360 μg/d MK-7 in patients with T2DM and pre-existing CVD did not decrease significantly systemic arterial calcification and calcification of femoral arteries [26, 27].

Different indexes have been developed to assess IR without a direct measure of insulin concentration. The indexes can be easily determined by routine measurements of FPG, triglycerides, and other lipoproteins. The indexes’ associations with subclinical atherosclerosis measurements of coronary artery calcification, arterial stiffness, and vascular flow limitations have been well indicated [5–9, 28]. The beneficial effects of an intervention on CVD risk by improving the metabolic variables of glucose and lipids can be exerted earlier by determining the indexes rather than atherosclerosis’s clinical measurements. Based on our findings, none of the indexes was significantly reduced following MK-7 supplementation for 12 weeks in our participants compared to the placebo. Previously, no studies examined the effects of vitamin K supplementation on atherogenic indexes.

Randomized double-blinded controlled design, high adherence to the study protocol, using the most effective and bioavailable forms of vitamin K are strengths of this study. The limitations of our study were: 1) the individuals’ responses to vitamin K supplementation may differ based on their vitamin K status before the supplementation. However, no measurement has been done for circulatory markers of vitamin K status in this study, so the participants’ vitamin K status was unclear at the beginning of the study; 2) we had no objective measurement to confirm the compliance of the study assessed by pill counting; 3) most participants were at the normal range of lipid profiles with/without lipid-lowering medications in this study. Therefore, they may have a lower response to the supplementation than those with abnormal lipid profiles. However, the AIP values of 84.1% of our participants were more than 0.21, suggesting that they were at a high risk of CVD [9]; 4) this study’s sample size is calculated based on the primary outcomes of glycemic variables, so it may not be adequately powered to detect changes in IR-related indexes; 5) testing multiple hypotheses simultaneously causes multiplicity concerns, which may increase the probability of false-positive findings [29, 30]. However, multiplicity adjustment was not considered in this study because of null findings; 6) the dose of vitamin K2 and the study duration was determined according to a study’s findings suggesting vitamin K status improvements following 360 μg/d MK-7 supplementations for 12 weeks [11]. However, this dose and duration may not be adequate to observe any significant effects on atherogenic status.

## Conclusion

In conclusion, our findings could not show any significant improvement in the atherogenic status of patients with T2DM as measured by IR-related indexes of CVD risk, including Castelli indexes, triglycerides/HDL-C ratio, AC, TyG-Index, AIP, METS-IR, and LCI, after 12-week supplementation of 360 μg/d MK-7. More clinical trials are needed to clarify the effects of MK-7 supplementation on laboratory or clinical markers of CVD in patients with T2DM.

## Data Availability

All data are presented within the manuscript. Raw data can be available by corresponding authors per reasonable request.

## Abbreviations

AC: Atherogenic coefficient
AIP: Atherogenic index of plasma
ANCOVA: Analysis of Covariance
BMI: Body mass index
CVD: Cardiovascular diseases
CVs: Coefficients of variations
Dp-ucMGP: Dephosphorylated-uncarboxylated MGP
FPG: Fasting plasma glucose
HbA1c: Glycated hemoglobin
HDL-C: High-density lipoprotein cholesterol
IR: Insulin resistance
IPAQ: International Physical Activity Questionnaires
LCI: Lipoprotein combine index
LDL-C: Low-density lipoprotein cholesterol
METS-IR: Metabolic score for insulin resistance index
MK-7: Menaquinone-7
MCP-1: *Monocyte Chemoattractant Protein-1*
MGP: Matrix Gla protein
SD: Standard deviation
SGLT2: Sodium-glucose co-transporter-2
T2DM: Type 2 diabetes mellitus
TyG-Index: Triglyceride-glucose index
VKDP: Vitamin K-dependent protein
